# Spatiotemporal and tissue-specific dynamics of bacterial communities in adult female *Ixodes ricinus* during a four-year period

**DOI:** 10.3389/fcimb.2026.1880365

**Published:** 2026-08-17

**Authors:** Sarah-Lena Heuser, Christian Zenner, Anna Wiesinger, Madlen Gänzle, Stephanie Hiereth, Philipp Girl, Klaus Neuhaus, Reinhard K. Straubinger

**Affiliations:** 1 Chair of Bacteriology and Mycology, Institute for Infectious Diseases and Zoonoses, Department of Veterinary Sciences, Faculty of Veterinary Medicine, Ludwig-Maximillian-Universität Munich, Oberschleißheim, Germany; 2 Clinic for Small Animal Surgery, Vetsuisse Faculty, University of Zurich, Zurich, Switzerland; 3 Core Facility Microbiome, ZIEL - Institute for Food & Health, Technische Universität München, Freising, Germany

**Keywords:** 16S rRNA gene amplicon sequencing, bacterial community, *Candidatus* Midichloria, spatiotemporal dynamics, *Ixodes ricinus*

## Abstract

**Introduction:**

The hard tick *Ixodes ricinus* serves as vector for life-threatening tick-borne diseases (TBDs) such as Lyme borreliosis, relapsing fever, and tick-borne encephalitis. Due to the ticks` ability to adjust to climate changes, their habitat is expanding quickly across Europe, and the incidences of TBD´s are increasing. Therefore, to better understand and control TBDs it is essential to expand the knowledge about the ticks´ microbiome, particularly the spatiotemporal and tissue-specific dynamics of *I. ricinus* bacterial community. This study was designed to investigate the differences in the detected bacterial communities according to the locations, where ticks had been collected, according to ticks´ tissue types and to seasonal impacts.

**Materials and methods:**

A total of 723 adult female *I. ricinus* were collected from two locations in Bavaria over a four-year period. A defined number of individual ticks were dissected; DNA was extracted from tissue samples or complete ticks, and subsequently 16S rRNA-gene amplicon sequencing was performed.

**Results and discussion:**

No clear impact of the seasons was detected across the complete four-year period. However, a yearly seasonal pattern was observed in spring (Sp) compared to autumn (Au). Particularly, Sp22, Sp23 and Sp24 showed no difference in effective richness. These results are consistent with previous studies. While the species richness changed between different locations, the effective richness did not. Specific tissue samples of the ticks recovered by dissection (salivary glands, midgut, exoskeleton) carried significant, distinct bacterial communities. The genus *Candidatus* Midichloria was most abundant and was detected in all tissue types. *Borrelia* spp. were not detected in any of the collected samples, besides in the mock communities, possibly due to limitations in 16S rRNA gene analysis.

## 1 Introduction

Ranking second after mosquitoes, ticks are important transmitters of infectious disease agents for human infections worldwide, making them a major threat for global health. Notably, ticks are also the primary carriers of pathogens that cause diseases in both domestic and wild animals (De La Fuente et al., 2008). Spanning around 900 species, ticks inhabit vast areas on our planet. To date, ticks are classified into three families: hard ticks (Ixodidae), soft ticks (Argasidae) and “intermediate” Nuttalliellidae (Schoch et al., 2020). In Germany, *Ixodes ricinus* is the most common and threatening tick species for human and companion animals and consequently, an eminent vector of TBDs (Robert-Koch-Institute, 2024).

Ticks occupy many different habitats, such as heathlands, urban parks, moorlands, forests, and rough grasslands. Their ability to colonize diverse environments is demonstrated by the appearance of *I. ricinus* in all geographic directions across Europe (Estrada-Pena, 2001; Estrada-Pena et al., 2013a; Medlock et al., 2013). Furthermore, rising environmental temperatures due to the climate change is fuelling the extent of their distribution (Semenza and Suk, 2018). These changes not only lead to regional shifts towards higher latitudes and higher altitudes, but also contribute to an increase of the total tick populations (Cunze et al., 2022; Medlock et al., 2013).

Ixodid ticks live through four developmental stages: from egg to larva to nymph, and finally to the imago, the final adult stage. A blood meal is required before each molt, hence, *I. ricinus* ticks usually undergo a three-host lifecycle. Their development takes between two and six years depending on temperature, seasonal conditions, and the availability of suitable hosts (Gray et al., 2002). A seasonal, biphasic activity rhythm characterizes their behaviour (Sonenshine and R., 2014). In Europe, their main questing activity takes place in spring and early summer when temperatures are around 10 °C. Nonetheless, it can extend from spring to autumn and often follows a bimodal pattern with peaks in spring and late summer/early autumn (Gray et al., 2002; Perret et al., 2000). However, variations in temperature, rainfall, sunshine, and relative humidity affects their activity (Gray et al., 2016). Climate change impacts the population on the long term. Thus, ticks benefit from warmer winters (temperatures above -10 °C) and humid summers (Lindgren et al., 2000; Semenza and Suk, 2018). Nevertheless, to survive harsh conditions of freezing winters and hot, dry summers, ticks enter diapause. This naturally occurring state of suspended development, which resembles apparent death, is hormonally regulated. Once diapause ends, the physiological functions return to their previous state (Bedzhov, 2020). By synchronizing development and host-seeking behaviour with the seasons, diapause helps minimize the risk of dehydration or freezing during dangerous periods (Gray et al., 2016). This remarkable ability to adapt to environmental conditions not only secures survival, but also shapes their role as disease vectors.

One of these critical diseases transmitted by *I. ricinus* ticks is Lyme borreliosis, which is caused by the bacterium *Borrelia burgdorferi* sensu lato, and is the most common vector transmitted infection in Germany. It is estimated that between 60,000 and 200,000 residents are infected with Lyme borreliosis each year (Wilking et al., 2023). Other common transmitted pathogens inducing TBDs include bacteria such as *Borrelia miyamotoi* (relapsing fever), *Coxiella burnetti* (Q fever), *Anaplasma phagocytophilum* (human granulocytic anaplasmosis), *Candidatus* Neoehrlichia mikurensis (neoehrlichiosis), *Francisella tularensis* (tularemia), parasites such as *Babesia microti*, and viruses such as tick-borne encephalitis virus, the most important tick-borne virus in Europe (Dobler et al., 2014; Medlock et al., 2013).

Although these pathogens are the primary focus of disease research, they represent only a small part of the broader tick microbiome. In this context, the microbiome plays a crucial role in understanding the tick physiology, shaping vector competence and pathogen transmission.

The microbiome not only encompasses the microbiota but also all molecules produced by microorganisms, such as metabolites, structural components, and coexisting host molecules (e.g. mobile genetic elements, phages, viruses, “relic” and extracellular DNA). The microbiota specifically refers to the community of all living organisms, including bacteria, fungi, algae, protists, and archaea (Whipps et al., 1988; Berg et al., 2020). Our focus lies, however, on the bacterial community (BC) present in the tick body (organs) and on the tick’s exoskeleton (residual tissue). Specific members of such bacterial communities can act as symbionts, endosymbionts, or may compete with pathogens (Aivelo et al., 2019).

Prior to the introduction of next generation sequencing (NGS), analysing and identifying components of the microbiome was a labour-intensive and time-consuming process. Traditionally, each bacterium or pathogen had to be identified individually using PCR or, in earlier methods, through culturing, staining, and microscopy, making comprehensive studies difficult.

Since the identification of the 16S rRNA gene in 1977 and the development of NGS, the entire bacterial community of e.g. ticks can be classified in a single step (Woese and Fox, 1977). The 16S rRNA gene encodes small subunit ribosomal RNA molecules of prokaryotic ribosomes and serves as the most widely used phylogenetic marker for bacteria (Yarza et al., 2014). After gene amplification and sequencing, the sequences are denoised and each such sequence (or very similar ones) are used for the characterization of the bacterial community (Yilmaz et al., 2014; Quast et al., 2013). Using this framework, the present study aimed to further expand the understanding of the ticks’ BC and to fill existing gaps especially by comparing locations, tissue types and seasons for the years 2021 to 2024.

## 2 Materials and methods

### 21 Tick sampling

Between autumn 2021 and autumn 2024, a total of 723 female *I. ricinus* ticks were collected continuously from two locations: Kranzberg, a large forest near Freising (FS) and Oberschleißheimer Schlosspark (OSH), a public park near Munich, South-Germany. Both locations are high-risk areas for tick-borne encephalitis and have a dense tick population (**Table 1**) (Robert-Koch-Institut, 2025). Organized local vector control programs were not implemented at either FS or OSH. The ticks were collected using the flagging method, in which a white sheet (1 × 1.5 m) was slowly dragged 2 to 3 minutes over meadows, woodlands, and vegetation and afterwards checked for tick presence (Dantas-Torres et al., 2013; Estrada-Pena et al., 2013b). Ticks were carefully removed with tweezers, placed separately into 1.5-mL reaction tubes (Eppendorf, Wesseling-Berzdorf, Germany) and brought to the laboratory within 2 h. Due to time constraints, ticks were generally not dissected directly after sampling. The tubes were either stored in the refrigerator and dissected the next day or immediately frozen at -20 °C for up to two-month (without added liquid) until processing. In the following, sample groups are given as Au, for autumn, or Sp for spring, and the respective year of sampling (e.g. 21 for 2021). Neither at the time of sampling nor at the time of dissection or DNA extraction the tick surfaces were sterilized. Tweezers for sampling were disinfected with DNA AWAY™ (Thermo Fisher Scientific, Waltham, MA, USA) before each sampling event and in between sampling sites.

**Table 1:** Summary of the prepared samples divided by locations and tissue at the end of the analysis (*) 3 tick organ samples were pooled.

Location	Complete tick CT	Salivary gland* SG	Mid gut* MG	Residual tissue* RE
Freising	192	48	59	51
Oberschleißheim	174	58	64	64
***Total***	***366***	***107***	***123***	***115***
	711			

*Total sum of both sampling locations divided by tissue type or complete tick.

Bold texts are the total sum of both sampling locations divided by tissue type or complete tick.

### 2.2 Dissection of female *I. ricinus* ticks

About 50% of the collected ticks, 345 of 711, were dissected. However, in Au2024 (Au24), only eleven ticks were collected, and no dissection was performed on the respective samples (**Table 1**). To improve sequencing quality, increase biomass input, and minimize the influence of contamination from reagents or environmental sources, all dissected tissue samples (i.e., salivary glands, midgut and the residual tissue) were pooled in groups of three (Gall et al., 2016; Ross et al., 2018). The dissection was conducted using a stereomicroscope with up to 120× magnification and a high depth of focus (Leica M205 C with FusionOptics, Leica Microsystem, Wetzlar, Germany). As necessary, frozen ticks were thawed and fixed to microscope slides using double-side tape universal (Tesa SE, Norderstedt, Germany). Tubes for three different tissue types, salivary gland (SG), midgut (MG) and residual tissue (exoskeleton; RE), were prepared filled with 100 µL of sterile-filtered phosphate-buffered saline (PBS, Carl Roth, Karlsruhe, Germany) each. A longitudinal incision was made from last leg on the right side to the last leg on the left using a scalpel blade (No. 15, Swann Morton, Sheffield, United Kingdom). Of note, a new sterile scalpel blade and microscope slide was used for each dissection. The incision was further extended above the first legs on both sides. The upper portion was lifted with tweezers (Dumont #5 Forceps 11252-20, Fine Science Tools, Heidelberg, Germany) and the internal tissue was exposed. Next, the MG tissue was carefully removed and placed into its designated tube (MG). Thereafter, the SG tissue were carefully extracted using a dental probe (Zahnsonde LS-Passion, Art.-Nr: LSP1081/33, Carl Martin, Solingen, Germany) and transferred to its respective tube (SG). The remaining tick structures, including the capitulum, idiosoma, legs, and residual tissue, were also placed in the RE tube. Dissected samples were stored at -30 °C until DNA extraction. All instruments and surfaces were disinfected with DNA AWAY™importantly after each contact with tick tissue.

### 2.3 DNA-extraction of complete and dissected ticks

For DNA extraction of complete ticks, the Maxwell 16 LEV Blood DNA Kit with the Maxwell 16 Instrument (both Promega, Walldorf, Germany) was used.

Complete ticks (CT samples; n = 366) were first mechanically cut multiple times using a scalpel blade (No. 15, Swann Morton Sheffield, United Kingdom) before being transferred into lysing matrix tubes (Lysing Matrix Tube D, 2.0 mL, MP Biomedicals, Eschwege, Germany). Subsequently, 300 µL of Incubation Buffer (D920B-C, Promega) were added. The samples were then processed in the FastPrep-24™ system (MP Biomedicals Germany, Eschwege) with a single homogenization cycle at 5.5 m/s for 30 s. Following homogenization, 30 µL of 20-mg/mL Proteinase K (AS1290, Promega) and 200 µL of Lysis Buffer (AS1290, Promega) were added. After mixing with a vortex mixer (Vortex-Genie 2, Scientific Industries, New York, NY, USA) and a brief centrifugation for 10 s at 9,391 × *g* (Centrifuge 5430, Eppendorf, Hamburg, Germany), the samples were incubated at 56 °C with continuous shaking at 350 rpm (ThermoMixer C, Eppendorf) for at least 2 h.

To degrade residual RNA, 5 µL of 10-mg/mL RNase A (Thermo Fisher Scientific, Waltham, MA, USA) were added, followed by an additional incubation at 37 °C and 350 rpm for 20 min. Afterwards, 300 µL of Lysis Buffer (Promega GmbH) were added, and vortexing, and centrifugation steps were repeated.

For dissected tick samples (n = 345), a similar procedure was followed. The samples were placed into tubes containing 300 µL of Incubation Buffer (D920B-C, Promega), followed by the addition of 30 µL of 20-mg/mL Proteinase K (AS1290, Promega) and 200 µL of Lysis Buffer (AS1290, Promega). The samples were then vortexed, centrifuged for 30 s at 9,391 × *g*, and incubated at 56 °C and 350 rpm for two hours. An additional 5 µL of 10-mg/mL RNase A (Thermo Fisher Scientific) were added, followed by a further incubation at 37 °C and 350 rpm for 20 min. Subsequently, 300 µL of Lysis Buffer (Promega GmbH) were added and the vortex mixing and centrifugation steps were repeated.

Negative controls were included in every extraction cycle to monitor potential contamination from dissection tools, reagents, and the Maxwell 16 MDx purification system. Afterwards, the complete *I. ricinus* ticks, dissected tick samples, and negative controls were processed using the automated Maxwell 16 MDx (Promega) system for DNA purification. The purified DNA was eluted in 60 µL Elution Buffer (from AS1290, Promega).

After extraction, DNA concentrations were measured using the BioPhotometer D30 (Eppendorf). Throughout all sampling years, the mean DNA concentration of a complete tick was 42.8 µg/mL. Samples were stored at -30 °C until shipping to the sequencing facility.

### 2.4 Design and creation of the mock community

Mock communities were designed to trace specific bacteria during data analysis (Abellan-Schneyder et al., 2021). Every sampling year included two different mock communities including the bacteria *Borrelia burgdorferi* sensu stricto*, Borrelia garnii, Borrelia afzelii, Borrelia bavariensis, Borrelia lusitaniae, Borrelia valaisana, Leptospira interrogans*, *Anaplasma phagozytophilum* and *Escherichia coli* in comparable concentrations. Further details about the design can be found elsewhere (Wiesinger et al., 2023).

### 2.5 16S rRNA gene amplicon sequencing

Ticks and negative controls from the years 2021 and 2022 were sequenced according to Wiesinger et al. (Wiesinger et al., 2023). Briefly, sequencing was performed using the same protocol, primer, and facility as for the 2023 samples, as described below. For DNA samples and negative controls from 2023, 20 µL each of extracted DNA was shipped on ice to Eurofins Genomics (Eurofins Genomics, Ebersberg, Germany) for sequencing of the 16S rRNA gene. Here the 16S gene was targeted using the primer given below in a two-step PCR. After amplicon clean-up, amplicons were sequenced on Illumina MiSeq. DNA samples and negative controls from 2024 were transported on ice to the Core Facility Microbiome (ZIEL – Institute for Food & Health, Freising, Germany) for sequencing. Amplicons were constructed targeting the variable regions V1-V3 of the 16S rRNA gene using the primers given below in in a two-step PCR (Reitmeier et al., 2020). After amplicon clean-up, amplicons were sequenced on Illumina MiSeq. The purified DNA of the years 2021, 2022 and 2023 was sequenced using primer 27F (5’-AGA GTT TGA TCC TGG CTC AG-3’) (Weisburg et al., 1991) and 530R (5’-GTA TTAC CGC GGC TGC TG-3’) (Leser et al., 2002). Only 2024 was sequenced using 27F and 534R (5’-ATT ACC GCG GCT GCT GG-3’) (Abellan-Schneyder et al., 2021). Both facilities used the same Illumina sequencing technology (Illumina, Berlin, Germany).

#### 2.5.1 Data analysis

Raw sequences were processed as follows. Primer sequences were removed due to length variability. Primer removal and a control script were performed using R scripts with the *Bioconductor* package *ShortRead* in R v4.3.3 using RStudio v2025.09.02 + 418. Next, by using the DADA2 pipeline, paired-end sequences were merged using the following parameters: truncLen = 260,220, maxN = 2,2, trunQ = 2, rm.phiX = true, and compress = true. Subsequently, the sequences were denoised, merged, and chimeras were removed (Callahan et al., 2016). Negative controls were included for quality assessment of potential contamination but were excluded from downstream microbial community analyses due to low sequencing depth.

Nine samples with less than 100 reads were excluded. Quality control was conducted using the Galaxy platform^1^ (Afgan et al., 2018) with FASTQC v0.12.1 (Andrews, 2010) and MultiQC v1.27 (Ewels et al., 2016). Sequencing depth was assessed on Amplicon Sequence Variant (ASV) reads prior to normalization. A total of three samples were excluded due to insufficient quality. Finally, an ASV table was constructed, and taxonomy was assigned using the rRNA gene reference database SILVA v138.2 (Yilmaz et al., 2014; Quast et al., 2013).

To use the Rhea pipeline^2^ (Lagkouvardos, 2024) for further analysis, data were converted in R package *phyloseq*. Rhea is an open-source pipeline including R-scripts for Normalization, *Alpha*-diversity, *Beta*-diversity, Taxonomic binning, Serial-Group-Comparison and Over-Time-Comparison. Prior to the *alpha*-diversity-analysis, the data were normalized, to avoid loss of information due to subsampling and an abundance filter of 0.25% was applied to remove low-abundance bacterial taxa, thereby reducing noise from rare or potentially spurious observations. This filtering step retained the biologically relevant fraction of the bacterial community and enabled more reliable comparisons among samples (Reitmeier et al., 2021). *Alpha*-diversity was calculated using effective richness and effective Shannon indices. As a result, all diversity indices reported represent effective diversity measures (Jost, 2006, 2007). *Beta*-diversity was determined by generalized UniFrac distances (Chen et al., 2012) and visualized by Multi-Dimensional Scaling (MDS) (Minchin, 1987). Statistically significant differences among groups (overall and pairwise) were calculated using permutational multivariate analysis of variance (PERMANOVA) on distance matrices implemented in the R-package *vegan* (function *adonis*) (Anderson, 2001). The homogeneity of group dispersions was assessed using permutational analysis of multivariate dispersions (PERMDISP) based on generalized UniFrac distances matrices, as implemented in the vegan package in R. Result from the Rhea scripts were further statistically analysed using Graph Pad Prism v5.04 (GraphPad Software, Boston, USA). Thereby the significance especially for the effective richness comparisons were assessed using the Kruskal-Wallis test followed by Dunn´s multiple comparisons test with adjustment for multiple testing. Significance level was defined as *p*-value ≤ 0.05. Visual representations were created using Graph Pad Prism and Affinity Designer 2.0 v2.6.3 (Serif, Nottingham, United Kingdom).

## 3 Results

Following 16S rRNA gene amplicon sequencing, primer removal, and quality assessment, each sample contained on average 60,366 reads/sample ± 42,186 raw reads/sample. After processing with the DADA2 pipeline, which included trimming and merging steps, the number of reads was reduced to a mean of 22,023 reads/sample ± 20,119 reads/sample.

**Figure 1:**
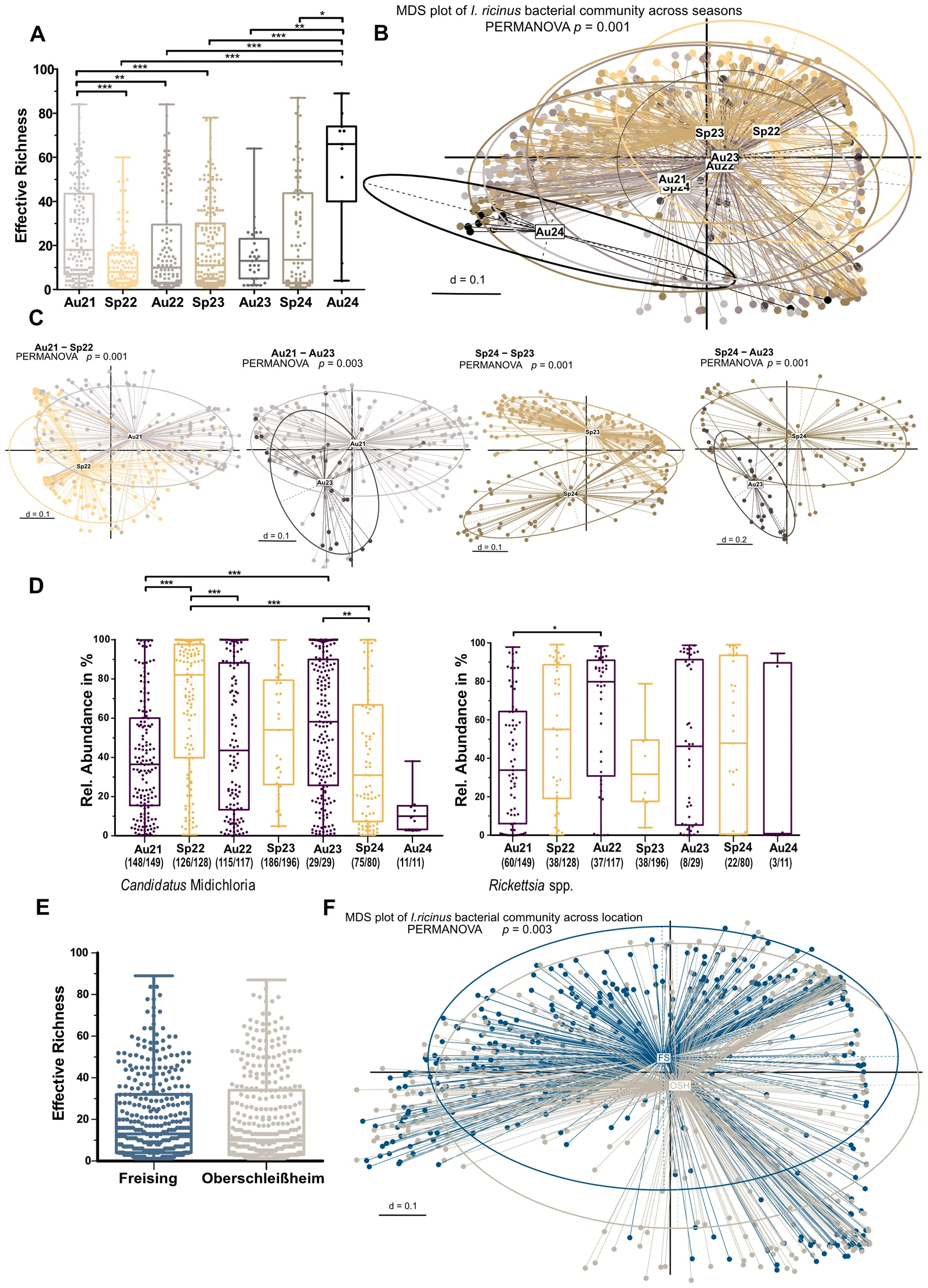
**(A)** Boxplots representing the effective richness across seasons: Au, autumn; Sp, spring.; *p* values were calculated by Kruskal-Wallis followed by a pairwise Dunn’s Multiple Comparison test and adjustment. *, *p* ≤ 0.05; **, *p* ≤ 0.01; ***, *p* ≤ 0.001. **(B)** Beta-diversity based on generalized UniFrac distances displayed as MDS ordination. p value was calculated by PERMANOVA. Au, autumn; Sp, spring **(C)** Pairwise beta-diversity comparison based on generalized UniFrac distances, displayed as MDS ordination. p values were calculated by PERMANOVA. Au, autumn; Sp, spring. **(D)** Boxplots representing the relative abundance (%) of *Ca*. Midichloria and *Rickettsia* spp. across seasons. *p* values were calculated by Kruskal-Wallis followed by a pairwise Dunn’s Multiple Comparison test and adjustment. *, *p* ≤ 0.05; ***: *p* ≤ 0.001; Prevalence displayed below the season (positive samples/number of all samples). **(E)** Boxplots representing the effective richness across locations (F, n = 350; OSH, n = 361). **(F)** Pairwise beta-diversity comparison based on generalized UniFrac distances, displayed as MDS ordination. p value was calculated by PERMANOVA. OSH, Oberschleißheim, FS, Freising.

Initial analyses of all samples revealed differences in the *alpha*-diversity across sampling years and seasons, measured by effective richness. The strongest differences were observed for Au21 vs. Sp22 (*p* ≤ 0.001), Au21 vs. Sp23 (*p* ≤ 0.01), Sp22 vs. Au24 (*p* ≤ 0.001), as well as for Au22 vs. Au24 (*p* ≤ 0.001). However, it should be noted that the number of samples for Au24 was limited (n = 11; **Figure 1A**).

Next, the *beta*-diversity of *I. ricinus* BC was assessed for all sampling time points (i.e., Au21, Sp22, Au22, Sp23, Au23, Sp24, and Au24) using generalized UniFrac as a distance metric. For easier visual comparison, so-called inertia ellipses are shown around each group, representing the variance and distribution of each group. A significant shift in the bacterial communities between seasons was observed (PERMANOVA, *p* = 0.001, F = 12.541, R^2^ = 0.097; PERMDISP, *p* = 0.001; **Figure 1B**).

Pairwise comparisons of *beta*-diversity between individual seasons revealed significant differences, including Au21 vs. Sp22 (PERMANOVA, *p* = 0.001, F = 24.01, R^2^ = 0.08; PERMDISP, *p* = 0.001), Au21 vs. Au23 (PERMANOVA, *p* = 0.003, F = 5.035, R^2^ = 0.028; PERMDISP, *p* = 0.001), Sp23 vs. Sp24 (PERMANOVA, *p* = 0.001, F = 19.427, R^2^ = 0.066; PERMDISP, *p* = 0.001), and Au23 vs. Sp24 (PERMANOVA, *p* = 0.001, F = 6.922, R^2^ = 0.061; PERMDISP, *p* = 0.001) (**Figure 1C**).

The relative abundance (%) of *Ca.* Midichloria and *Rickettsia* spp. was investigated. To see differences between seasons, all tissue types were combined. Indeed, significant differences between the years were observed for *Ca.* Midichloria for Au21 vs. Au23 (*p* ≤0.001), Au22 vs. Au23 (*p* ≤ 0.001), Sp23 vs. Sp24 (*p* ≤ 0.001), and Au23 vs. Au24 (*p* ≤ 0.001). Of note, as before, the number of samples in Au24 was limited (n = 11). For *Rickettsia* spp., only one significant difference in relative abundance was observed for Au21 vs. Au22, *p* ≤ 0.05. The prevalence of both bacterial genera is shown in **Figure 1D**.

Following the analysis of temporal variation, the BC composition was analysed across the different sampling locations. The *alpha*-diversity, measured as effective richness, did not differ between the two locations compared, FS and OSH (**Figure 1E**). In contrast, ticks from the two locations differed in their *beta*-diversity (PERMANOVA, *p* = 0.003, F = 5.035, R^2^ = 0.028; PERMDISP, *p* = 0.001), as can be seen in the MDS-plots in **Figure 1F**.

**Figure 2:**
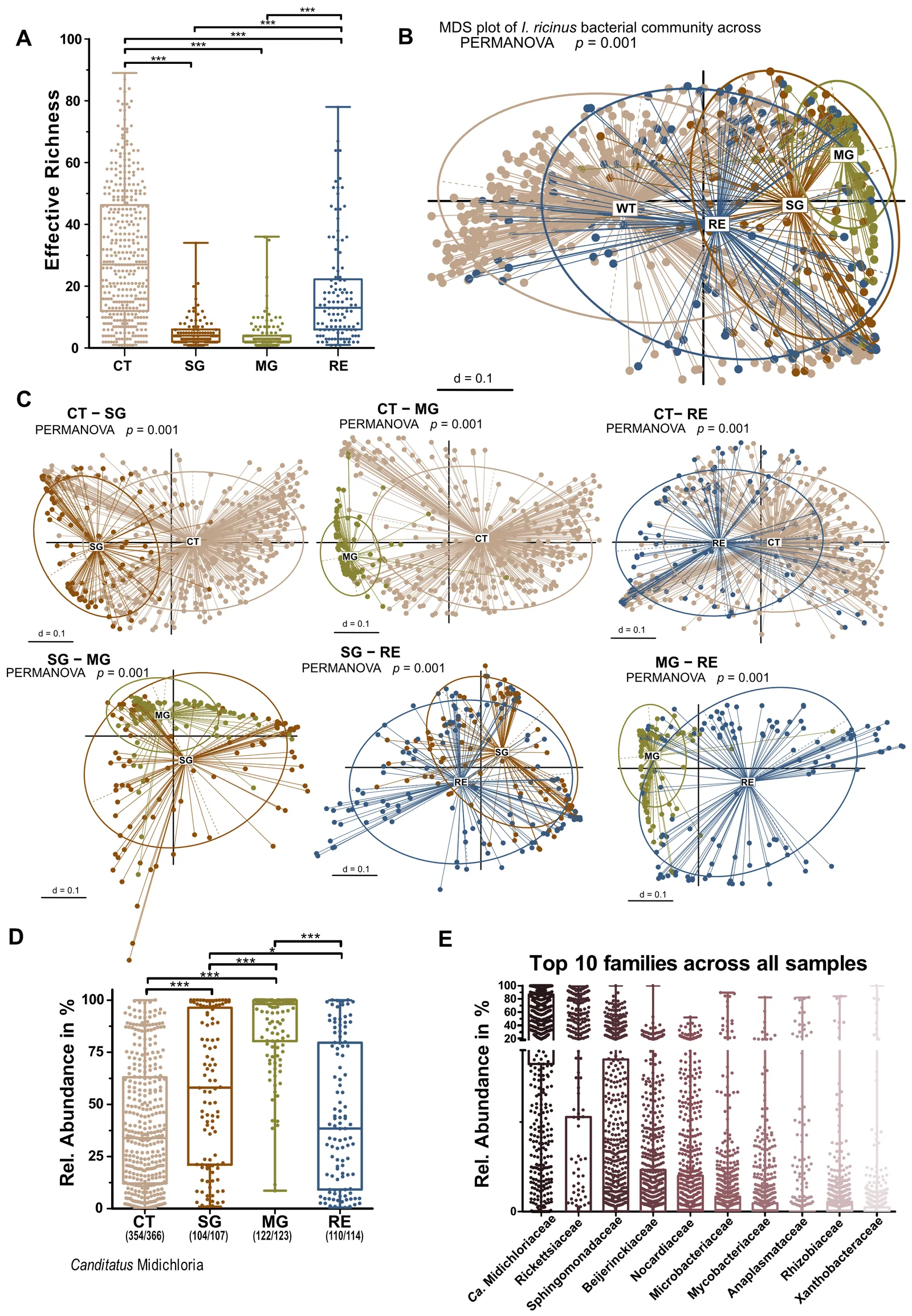
**(A)** Boxplots representing the effective richness across different tissues; p values were calculated by Kruskal-Wallis followed by a pairwise Dunn’s Multiple Comparison test and adjustment. ***, *p* ≤ 0.001. **(B)** *Beta*-diversity displayed as MDS ordination based on generalized UniFrac distances. *p* value was calculated by PERMANOVA. CT, complete tick; SG, salivary gland; MG, midgut; RE, residual tissue. **(C)** Pairwise beta-diversity comparisons displayed as MDS ordination based on generalized UniFrac-distances. *p* values were calculated by PERMANOVA. CT, complete tick, SG, salivary gland; MG, midgut; RE, residual tissue. **(D)** Boxplots representing the relative abundance (%) of *Ca*. Midichloria compared across tissues. *p* values were calculated by Kruskal-Wallis followed by a pairwise Dunn’s Multiple Comparison test and adjustment. *, *p* ≤ 0.05; ***, *p* ≤ 0.001; prevalence is given below the tissue name (positive samples/number of all samples). **(E)** Boxplots representing the relative abundance (%) of the top 10 bacterial families with the highest abundance. p values were calculated by Kruskal-Wallis followed by a pairwise Dunn’s Multiple Comparison and adjustment. To improve data visualization, the y-axis was divided into two sections, first from 0 - 18%, and 18% - 100%.

For analysing the bacterial communities of individual organs, collected ticks were dissected into SG, MG, and RE. Furthermore, some complete ticks were also assessed (see Methods). For the dissected parts, the *alpha*-diversity was measured as effective richness, and significant variations were observed between all pairwise combinations tested (*p* ≤ 0.001), except for SG vs. MG. Notably, the effective Shannon numbers showed significant differences across all comparisons, including SG vs. MG (**Figure 2A**).

Similarly, as before, differences in *beta*-diversity were determined by generalized Unifrac distances, now of the tissue types, and were evaluated using MDS-plots. For comparison of all different tissue types, significant differences were observed between at least one of the groups (PERMANOVA, *p* = 0.001; F = 44.76; R^2^ = 0.16; PERMDISP, *p* = 0.001; **Figure 2B**).

Next, the pairwise comparisons were assessed to determine, which group caused the significant difference in the overall comparison. Notably, all tissue types differed from each other in their *beta*-diversity (PERMANOVA, *p* = 0.001 for all comparisons, CT vs. SG (PERMANOVA, *p* = 0.001, F = 38.62, R^2^ = 0.146; PERMDISP, *p* = 0.001), CT vs. MG (PERMANOVA, *p* = 0.001, F = 83.381, R^2^ = 0.146; PERMDISP, *p* = 0.001), CT vs. RE (PERMANOVA, *p* = 0.001, F = 12.699, R^2^ = 0.026; PERMDISP, *p* = 0.001), SG vs. MG (PERMANOVA, *p* = 0.001, F = 14.413, R^2^ = 0.059; PERMDISP, *p* = 0.001), SG vs. RE (PERMANOVA, *p* = 0.001, F = 12.427, R^2^ = 0.054; PERMDISP, *p* = 0.001), MG vs: RE (PERMANOVA, *p* = 0.001, F = 44.763, R^2^ = 0.16; PERMDISP, *p* = 0.001; **Figure 2C**).

In addition, **Figure 2D** presents the association of tissue type and the bacterium *Ca.* Midichloria. This bacterium was detected in all tissue types with differing, but overall high prevalence. Some samples even showed a relative abundance of 100% for *Ca.* Midichloria, thus, any of the other bacteria were either not present or below detection level (up to 100% relative abundance for the highest sample see **Figure 2D**).

Finally, **Figure 2E** depicts the top 10 most abundant families across all samples. Here *Ca.* Midichloriaceae represented generally the highest relative abundance, followed by Rickettsiaceae, Sphingomonadaceae, Beijerinckiaceae, Nocardiaceae, Microbacteriaceae, Mycobacteriaceae, Anaplasmataceae, Rhizobiaceae, and Xanthobacteraceae (**Figure 2E**).

## 4 Discussion

The presented study provides insights into the BC of *I. ricinus*, including its regional distribution, its distribution across different tick tissues and temporal changes during a four-year period. In 2021, Lejal et al. hypothesized that specific components of the microbiota of *I. ricinus* exhibit interannual recurrence, as reflected by their relative abundance. In other words, similar bacterial communities are observed during the same seasons across different years (Lejal et al., 2021). In contrast, White et al. did not report any such interannual recurrence of the microbiota in abundance in the UK (White et al., 2025). In line with the latter, effective richness was similar only between Sp22, Sp23, and Sp24 (**Figure 1A**), while the effective richness observed in autumn indicated significant differences between the years, i.e., Au21, Au22, Au23 and Au24 (**Figure 1A**). However, the *beta*-diversity analyses further demonstrated significant temporal differences in overall community composition (PERMANOVA, R² = 0.097, p = 0.001), indicating that bacterial community structure varies across seasons and years. PERMDISP analyses also revealed significant differences in within-group dispersion (*p* = 0.001), suggesting that seasonal differences are associated not only with shifts in community composition but also with changes in community heterogeneity. Nevertheless, this pattern was supported by the MDS ordination, which showed distinct clustering of samples according to season. Thus, the hypothesis of interannual recurrence of the microbiota proposed by Lejal et al. is not strongly supported by our *beta*-diversity results (Lejal et al., 2021). In spring, following the challenging winter period, there could be a higher proportion of newly emerged adults with a less developed BC. Alternatively, the BC of the existing ticks may need to regain their complexity during summer.

After detecting seasonal differences in the BC of *I. ricinus*, regional variations should also be assessed. The locations OSH and FS are in the same administrative region, Upper Bavaria, Germany, and are not in close proximity to the river Isar. The collection sites are roughly 28 km apart. OSH is a cultivated park near the castle of Schleißheim, mainly inhabited by small mammals, and also characterized by many visits of dog walkers. In contrast, FS is a mixed forest with a greater proportion of large mammals (e.g., deer, boar, fox, mustelids), and a more natural forest vegetation. Variations in forest floor structure, vegetation type, and host animal species may influence the BC of ticks (Carpi et al., 2011; Kirstein et al., 1997; Van Overbeek et al., 2008; Krawczyk et al., 2022; Wielinga et al., 2006). Therefore, different results are expected in both regions. While the effective richness (number of taxonomic units found) was similar, the *beta*-diversity analysis revealed significantly different community compositions between locations (**Figures 1E, F**). However, the effect size was small (R^2^ = 0.006), suggesting that geographic location had only a minor influence on the overall BC structure. Furthermore, PERMDISP analyses did not indicate differences in within-group dispersion (*p* = 0.752), suggesting that the observed differences are attributable to shifts in community composition rather than differences in community heterogeneity. Although the literature is not conclusive concerning differences in locations, there is a tendency towards deviations. While White et al. did not find statistically significant differences between woodland types (e.g., pine or oak), Van Overbeek et al. and others reported such differences between three forest sites in the Netherlands, that are 100 to 166 km apart (Van Overbeek et al., 2008). Furthermore, Wielinga et al. even observed differences in bacterial diversity between locations that were only 300 m apart (Wielinga et al., 2006).

While external factors, such as sampling locations, represent only one component of the BC of *I. ricinus*, internal factors should not be ignored. Significant differences in effective richness were observed between most tissue types. The species richness of RE was significantly higher compared to internal tissues (SG and MG; **Figure 2A**). However, RE tissue is not the primary contributor to the BC of *I. ricinus*. This is demonstrated by the significant difference between RE and CT in **Figure 2A**, confirming that the BC is jointly shaped by internal and external compartments (Berg et al., 2020). Only the internal tissues, SG and MG, expressed no variation in species richness (**Figure 2A**), which may not be surprising given the challenge of dissection. SG and MG showed no difference in *alpha*-diversity, whereas *beta*-diversity analyses revealed significant difference in BC composition, suggesting that these tissues exhibit similar levels of diversity but differ in their BC structure. An effect size ranging from R^2^ = 0.026 to 0.16 suggests that tissue type plays a relevant role in shaping BC composition. In addition, PERMDISP analyses indicated that the variability of bacterial communities differed between tissues. (**Figure 2C**). Notably, MG samples showed more distinct clusters in the MDS-plots compared to the other groups, indicating lower variation of bacterial species within MG samples (**Figure 2C**).

To further explore this, the distribution in tissues of the most abundant bacterium in *I. ricinus*, *Ca.* Midichloria, was analyzed. *Ca.* Midichloria was detected in all examined tissues with a prevalence of SG 97.2%, MG 99.2%, and RE 96.5%. The relative abundance in SG was more widely dispersed compared to MG. MG almost exclusively showed high relative abundance samples, which is likely because *Ca.* Midichloria is primarily found in the ovaries of *I. ricinus* according to literature (Olivieri et al., 2019; Sassera et al., 2006). Nevertheless, the tissue-based analysis suggest that *Ca.* Midichloria is not exclusively located in ovaries. This is supported by the finding that *Ca.* Midichloria was detected in 44% of male ticks via PCR (Lo et al., 2006). The variation in relative abundance of *Ca.* Midichloria in RE tissue might be explained by the fact that RE tissue was not treated with DNA AWAY™ after dissection; consequently, parts of MG and SG may have remained in the sample. Alternatively, *Ca.* Midichloria might be present on the external surface of ticks. The tissue-specific distribution of *Ca.* Midichloria provides important insights into its potential functional role. Yet, it remains unclear whether these patterns are stable over time or subject to seasonal variation. Remarkably, a similar seasonal pattern was observed for the intramitochondrial endosymbiont *Ca.* Midichloria, as mentioned above for the entire BC. Notably, the relative abundance of *Ca.* Midichloria differs significantly between the consecutive seasons of Au21, Sp22 and Au22 and then only between the two consecutive seasons Au23 and Sp24 (**Figure 1D**). A study by Hartemink et al. found a clear pattern for *Ca.* Midichloria mitochondrii, with an increase from spring to summer and a decrease from summer to autumn (Hartemink et al., 2024). However, they collected nymphs monthly over four years and used qPCR for measurement, in contrast to the seasonally collected adult female ticks analysed by 16S rRNA sequencing in this study. Nevertheless, the vertically transmitted bacterium *Ca.* Midichloria has been reported to have a prevalence of up to 100% in female *I. ricinus* (Lo et al., 2006; Sassera et al., 2006). Indeed, the prevalence of *Ca.* Midichloria observed in this study was 97.2% across all tissues, which is consistent with the previous findings. Some negative samples might be explained by inadequate dissection. *Ca.* Midichloria is among the most prevalent genera in *I. ricinus*, and it also belongs to the most abundant family, *Ca.* Midichloriaceae, followed by Rickettsiaceae and Sphingomonadaceae.

Similarly to Lejal et al., the two most abundant families (*Ca.* Midichloriaceae and Rickettsiaceae) were also represented within their top four genera, whereas *Arsenophonus* was not detected and *Wolbachia* was detected in six samples. Both are associated with the parasitic wasp *Ixodiphagus hookeri* in ticks, but their primary targets are nymphs (Krawczyk et al., 2022; Lejal et al., 2021). Considering that Lejal et al. only tested nymphs and not adult ticks, it is more likely that they detected *Arsenophonus* and *Wolbachia* (Lejal et al., 2021; Sarda Carbasse et al., 2025).

Further bacterial families with the highest abundances across all samples included Sphingomonadaceae, Beijerinckiaceae, Nocardiaceae, Microbacteriaceae, Mycobacteriaceae, Anaplamataceae, Rhizobiaceae, and Xanthobacteraceae. The representatives of the families Anaplasmataceae and Rickettsiaceae are well known bacterial taxa associated of *I. ricinus* (Lejal et al., 2021).

Similar to Midichloriaceae, members of the family Rickettsiaceae, particularly those belonging to the *Rickettsia* spp., are likely to function as endosymbionts in ticks. Certain species, such as *R. helvetica*, are also pathogenic to humans (Krawczyk et al., 2022; Greay et al., 2018). Since *Rickettsia* spp. represent the second most abundant genus in *I. ricinus*, they are another suitable candidate for investigating potential seasonal recurrence. In the case of the relative abundance of *Rickettsia* spp., only minor differences between seasons were detected, with almost no significant variation observed (**Figure 1D**). This, together with the prevalence, indicates a relatively consistent occurrence across seasons and further supports the hypothesis of temporal recurrence.

The wide range of relative abundance of both *Ca.* Midichloria and *Rickettsia* spp. among samples may be explained by differences in sample composition, as some samples consisted of pooled dissected tissue (SG, MG, and RE), whereas others consisted of complete ticks. For example, MG samples are more likely to contain higher abundances of *Ca.* Midichloria, whereas RE samples may contain lower abundances.

In contrast to the aforementioned potential pathogen *Rickettsia* spp.*, Borrelia* spp. are frequently described as pathogenic (Gray et al., 2002; Nau et al., 2009; Philipp et al., 2001; Hoffmann et al., 2021). Medlock et al. demonstrated that the prevalence of *Borrelia burgdorferi*, an important pathogen within the genus *Borrelia* spp., varied over four-years period without a clear recurring pattern (Medlock et al., 2022). Interestingly, in our analysis, we were unable to detect any *Borrelia* spp., in any of the tick samples, while we generally could detect this genus in the mock communities. We even reanalysed the data using denoising into zero-radius Operational Taxonomic Unit (and not ASV) and analysis with the TAC algorithm (Lagkouvardos et al., 2016; Kioukis et al., 2022) but again we could not find meaningful sequences specific for the genus *Borrelia*. Thus, despite having a mean of 20k-reads for the samples, *Borrelia* were either not present or below detection level (0.0001% relative abundance), since highly abundant bacteria can mask spurious bacteria with low abundance (Gloor et al., 2017). In any case, White et al. also could not detect *Borrelia* spp. in 2021 (White et al., 2025). Still, it is unlikely that ticks in the sampling locations were not infected with *Borrelia* spp., emphasized by a prevalence study in western Bavaria, Germany, with a 12.7% prevalence of *Borrelia burgdorferi* sensu lato in *I. ricinus* (Zubriková et al., 2020). Moreover, an improved approach to detected *Borrelia* spp., a causative agent of Lyme borreliosis, could involve qPCR and 16S rRNA sequencing of both the pathogen-infected tick and infected host (Hoffmann et al., 2021).

In addition to identifying meaningful patterns, these results should be interpreted considering several limitations. One important limitation of this study is the unequal sample sizes across years. Due to environmental influences, the tick density varied considerably on both locations. One potential environmental factor is the unknown exposure to acaricides, through informal use by dog owners, which can have negative consequences on the tick population. For instance, in autumn 2024, only 11 ticks were found and not dissected. Obviously, dissection of the ticks is challenging, and variabilities cannot be excluded. Another limitation might be the use of slightly different primer sequences due to a change in sequencing facilities. The reverse-primer differed by an offset of four bases, thus, differences caused by primer bias cannot be fully excluded. In general, 16S rRNA sequencing allows classifications at the genus level. For the V1-V3 regions chosen here, about one third of the sequences can even be placed within the species (Soriano-Lerma et al., 2020). Contradicting, previous work by Chandra et al. and Rodino et al. suggests that the V1-V3 region provides improved detection of *Borrelia* spp. (Rodino et al., 2021; Chandra et al., 2021).

16 rRNA gene sequencing struggles with comparability, this is why the already sequenced data from 2021 and 2022 defined the target region (Durazzi et al., 2021; White et al., 2025). In the future, targeted approaches, like qPCR for *Borrelia* spp. might be more suitable.

In general, stricter standardization of methodologies used for microbiome studies in ticks and particularly for *I. ricinus* should be considered, since technical variability introduces bias. To improve comparability, methodological aspects such as sampling techniques, sequencing methods, analysis methods, and target regions should be standardized. With the absence of standardization, researchers may need to perform steps repeatably for comparisons, which slows down the overall process of understanding the microbiome of *I. ricinus* (Yilmaz et al., 2011).

A further limitation is that several low-abundance ASVs were detected in the negative controls. These ASVs were assigned to bacterial genera commonly associated with environmental sources such as soil, water, plants, or animals, including *Methylorubrum, Methylobacterium, Afipia, Microbacterium* and *Paracoccus.* All the negative controls with low sequencing depth, defined as insufficient read counts after quality filtering, were excluded from downstream analyses to ensure comparability. Although the remaining negative controls contained only low read counts, a complete exclusion of the background-derived sequences cannot be guaranteed. Since the present study also aimed to capture the environmental fraction of the *I. ricinus* BC, these ASVs were not removed from the ASV table and were therefore retained for downstream analyses. Consequently, low-abundance taxa should be interpreted with caution.

In summary, the findings of this study – including (1) an inconclusive seasonal recurrence pattern, (2) differences in BC between two locations, (3) higher effective richness in RE and CT compared to SG and MG, and (4) the high abundance of *Ca.* Midichloria in all tissue types, along with a seasonal pattern similar to that of the overall BC – contribute to the current understanding of the BC associated with *I. ricinus* and underscore the need for further research. Additional tick sampling at multiple locations – both closely situated and widely separated – across Bavaria or even Germany, along with extended study periods and more comprehensive data analyses, is necessary to thoroughly evaluate the hypothesis of interannual recurrence and local variation. Long-term studies spanning up to ten or more years may be required to reliably assess recurrence patterns.

## Data Availability

The datasets presented in this study can be found in online repositories. The names of the repository/repositories and accession number(s) can be found below: https://www.ebi.ac.uk/ena, PRJEB111026.

## Footnotes

1 https://usegalaxy.org.

2 https://github.com/Lagkouvardos/Rhea.
